# Neutrophil-to-lymphocyte ratio, platelets-to-lymphocyte ratio, and eosinophils correlation with high-resolution computer tomography severity score in COVID-19 patients

**DOI:** 10.1371/journal.pone.0252599

**Published:** 2021-06-28

**Authors:** Milena Adina Man, Ruxandra-Mioara Rajnoveanu, Nicoleta Stefania Motoc, Cosmina Ioana Bondor, Ana Florica Chis, Andrei Lesan, Ruxandra Puiu, Sergiu-Remus Lucaciu, Elena Dantes, Bianca Gergely-Domokos, Ovidiu Fira-Mladinescu

**Affiliations:** 1 Department of Medical Sciences, Pulmonology, "Iuliu Hatieganu" University of Medicine and Pharmacy, Cluj Napoca, Cluj, Romania; 2 "Leon Daniello," Clinical Hospital of Pulmonology, Cluj Napoca, Cluj, Romania; 3 Department of Medical Biostatistics, "Iuliu Hatieganu" University of Medicine and Pharmacy, Cluj Napoca, Romania; 4 Faculty of Medicine, "Ovidius" University, Constanta, Romania; 5 Center for Research and Innovation in Personalized Medicine of Respiratory Diseases, Department of Infectious Diseases, Pulmonology, “Victor Babes” University of Medicine and Pharmacy, Timisoara, Romania; All India Institute of Medical Science - Bhopal, INDIA

## Abstract

Inflammation has an important role in the progression of various viral pneumonia, including COVID-19. Circulating biomarkers that can evaluate inflammation and immune status are potentially useful in diagnosing and prognosis of COVID-19 patients. Even more so when they are a part of the routine evaluation, chest CT could have even higher diagnostic accuracy than RT-PCT alone in a suggestive clinical context. This study aims to evaluate the correlation between inflammatory markers such as neutrophil-to-lymphocyte ratio (NLR), platelets-to-lymphocytes ratio (PLR), and eosinophils with the severity of CT lesions in patients with COVID-19. The second objective was to seek a statically significant cut-off value for NLR and PLR that could suggest COVID-19. Correlation of both NLR and PLR with already established inflammatory markers such as CRP, ESR, and those specific for COVID-19 (ferritin, D-dimers, and eosinophils) were also evaluated. One hundred forty-nine patients with confirmed COVID-19 disease and 149 age-matched control were evaluated through blood tests, and COVID-19 patients had thorax CT performed. Both NLR and PLR correlated positive chest CT scan severity. Both NLR and PLR correlated positive chest CT scan severity. When NLR value is below 5.04, CT score is lower than 3 with a probability of 94%, while when NLR is higher than 5.04, the probability of severe CT changes is only 50%. For eosinophils, a value of 0.35% corresponds to chest CT severity of 2 (Se = 0.88, Sp = 0.43, AUC = 0.661, 95% CI (0.544; 0.779), p = 0.021. NLR and PLR had significantly higher values in COVID-19 patients. In our study a NLR = 2.90 and PLR = 186 have a good specificity (0.89, p = 0.001, respectively 0.92, p<0.001). Higher levels in NLR, PLR should prompt the clinician to prescribe a thorax CT as it could reveal important lesions that could influence the patient’s future management.

## Introduction

Coronavirus disease 2019 (COVID-19) is a rapidly progressive and sometimes fatal infectious disease caused by severe acute respiratory syndrome coronavirus 2 (SARS-CoV-2) [1, 2]. Described for the first time in China (Wuhan city, Hubei province) at the end of 2019, it was declared, in March 2020, a pandemic by the World Health Organization [1].

Infectious diseases are associated with inflammation, and existing data supports its significant role in the progression of various viral pneumonia, including COVID-19. Cellular destruction, because of SARS-COV2 viral replication, leads to cytokines and chemokines from the activated macrophages. Therefore they activate immune responses, leading to cytokine storms and aggravations. The severity of the inflammatory response determines the adaptive immune response, resulting in immune response imbalance. Procalcitonin (PCT), serum ferritin, erythrocyte sedimentation rate (ESR), C-reactive protein (CRP), and interleukin-6 (IL-6) have been associated with high risks for severe COVID19 infection [3–5]. Circulating biomarkers that can offer information about inflammation and immune status are potentially useful in the diagnosing prognosis of COVID-19 patients. The neutrophil-to-lymphocyte ratio (NLR) and platelet-to-lymphocyte ratio (PLR) are indicators of the systematic inflammatory response [6]. They have been widely investigated in several other conditions such as malignancies (included hematological malignancies), respiratory, gastrointestinal, cardiovascular (acute coronary syndrome, intracerebral hemorrhage) or s, systemic diseases. Higher values have been associated with more severe forms of illness with the worst prognosis [7–9].

As they are part of the routinely evaluated blood test, they are inexpensive, easily measurable, and widely available; they have also been assessed in COVID-19 patients. Higher values seem to be associated with more severe forms of the disease and more intensive care admissions. Although several benefits have been proposed, there is still no cut-off from which a severe form of the disease could be suspected [10]. Eosinophils are potent pro-inflammatory cells, and recent studies on mice have highlighted the fact that they have pleiotropic roles as they are involved in protective immunity, including antiviral responses and shaping diverse physiological responses) [11, 12]. Their peripheral blood level decreased dramatically, sometimes reaching zero in patients with COVID-19 related pneumonia compared with other types of pneumonia. When combined with NLR, they have a higher predictive value and could help in COVID-19 diagnostic and risk stratification, as they tend to be diminished from the very beginning [13]. Computer tomography of the chest has an essential role in the diagnosis and management of COVID-19 patients. It can help in patients’ trials- suggesting alternative diagnoses and evaluating the extent of disease, predicting worsening or improvement [14, 15].

In a suggestive clinical context, chest CT could have even higher diagnostic accuracy than RT-PCR alone. A low viral load may give a falsely negative test, while chest CT showed suggestive abnormalities even in asymptomatic patients [16–18]. Some of these patients may have a normal CT appearance initially, and the lesions appear afterward. COVID-19 is an infectious disease with significant systemic inflammation. There are some notable hematological changes such as leucopenia, lymphopenia, eosinopenia, elevated CRP, ESR. Some changes are specific for SARS-COV 2 infection, like elevated D-dimers, high ferritin, and high LDH levels [1–10]. COVID-19 is more contagious than seasonal flu, and specific populations (older people and those with comorbidities, especially cardiovascular comorbidities) have a higher risk of death from COVID-19.

Nevertheless, even younger people without underlying conditions may develop severe and sometimes lethal complications, such as fulminant myocarditis or disseminated intravascular coagulopathy. SARS-CoV-2 tends to affect tissues expressing high levels of ACE2, including the lungs, heart, and gastrointestinal tract. Looking for clinical and laboratory tests that can suggest a possible poor outcome is an important task.

The purpose of the present paper was to evaluate the correlation between inflammatory markers such as neutrophil-to-lymphocyte ratio (NLR), platelets-to-lymphocytes ratio (PLR), and eosinophils (EO) with the severity of CT lesions in patients with COVID-19. The second objective was to seek a statically significant cut-off value for NLR and PLR that could suggest COVID-19. Correlation of both NLR and PLR with already established inflammatory markers such as CRP, ESR, and those specific for COVID-19 (ferritin, D-dimers, and eosinophils) was also evaluated.

## Material and methods

The present paper was a cross-sectional study that took place in a Tertiary Pulmonology hospital, from the biggest city in the western part of Romania, a first-line hospital in the battle against COVID-19.

### Study population

The study included all patients with confirmed SARS-COV 2 infection consecutively hospitalized between March 27, 2020, till June 30, 2020. COVID-19 diagnostics were confirmed using real-time reverse-transcriptase polymerase-chain-reaction (RT-PCR) assay to test nasal and pharyngeal swab specimens according to WHO guidance. In Romania, hospitalization was compulsory for all patients diagnosed with COVID-19 regardless of the disease’s clinical severity. Therefore, the patients were asymptomatic, had a mild, moderate, or severe form of the disease. A healthy age-matched control group was included for comparison. Inclusion criteria consisted of all hospitalized patients over 18 years old with confirmed COVID-19 infection. Exclusion criteria: patients with confirmed COVID- 19 and with other comorbidities such as cancer, hematological diseases, severe cardiac disease (NYHA III and IV cardiac failure, recent myocardial infarction -last three months, unstable arrhythmia), liver disease, systemic diseases, and pulmonary fibrosis. The same number of age-matched healthy subjects were taken for comparison. The control group was clinically examined and had blood tests performed.

### Study design

Demographic, clinical, laboratory, and treatment data were taken from the patients at admission and extracted from electronic medical records. Laboratory tests were collected from all the patients at entry in the hospital (before any intervention) and were recorded. NLR ratio was calculated as the absolute count of neutrophils divided by the total count of lymphocytes. The PLR was defined as the absolute count of platelets divided by the absolute count of lymphocytes. Blood examinations involved measuring complete blood cell count and differential values. Serum biochemical tests, erythrocyte sedimentation rate, C-reactive protein, procalcitonin, D-dimers, and serum ferritin were done for COVID-19 patients. The control group had complete blood cell count and differential values, serum biochemical tests, erythrocyte sedimentation rate, and C-reactive protein. All laboratory tests were done in the hospital laboratory with standard procedures. The laboratory reference values of white blood cells, neutrophils, lymphocytes, eosinophils were 4.2–10, 1.8–7.3, 1.5–4, and 0.05–0.35 *103/ul, respectively. For ferritin was 30-220ug/la, and D-dimers were considered positive if they were above 500 ng/ml FEU (25- 5000ng/ml FEU). For procalcitonin, a value above 0.5 ng/ml was considered suggestive of bacterial infection. High-resolution computer tomography (HRCT) of the chest was performed in all patients in the hospital radiology department, and the images were afterward reviewed by a radiologist and a pulmonologist (the same persons throughout the entire study). The study was approved by the Hospital Ethics Committee nr 202/21.03.2020. All patients age over 18 years old gave a written and a verbal informed consent. The verbal consent was given by the patients in the presence of one doctor and one nurse after completely understanding every procedure. Afterwards the written consent was signed and sent via email to the doctors.

Typical imaging findings in COVID-19 patients include ground-glass opacities (GGO) with peripheral and subpleural distribution, usually involving lower lobes. All the lesions were analyzed based on sites and sizes. The lesion size was described as small (diameter, < 1 cm), medium (diameter, 1 to < 3 cm), or large (diameter, ≥3 cm). The CT images were also analyzed for a peripheral or central location, subpleural, ground-glass opacity (GGO) or consolidation, a halo sign of ground glass around a solid nodule, supply pulmonary artery dilation, air bronchogram, interstitial thickening, and other abnormalities (pleural effusion, cavitation, lymphadenopathy, etc.). The lesion was considered peripheral if it was located outer one-third of the lung and central for the other locations. The lesion area’s supply pulmonary artery was compared pulmonary supply artery of the lesion area with other pulmonary arteries at the same or similar normal segment. Considering the GGO areas or the size of the lesions, we divided the patients into three severity stages: 1 or mild severity less than three areas of 3 cm or ≥ 3 cm maximum diameter, 2. ≤ 3 GGO areas or ≤ 3 cm maximum diameter, or multiple lung areas of GGO associated with a tendency to lung consolidation(<50% of lung parenchyma)–moderate severity and 3—diffuse GGO or lung consolidation(> 50% of lung parenchyma) with signs of distortion of the lung architecture–high severity. The above CT terminologies were described using the international standard defined by the Fleischner Society Glossary.

### Statistical analysis

It was performed using IBM SPSS STATISTICS 25.0 application. Median (25th percentiles; 75th percentiles) was calculated for quantitative variables with a non-normal distribution; arithmetic means standard deviation was calculated for quantitative variables with a normal distribution. Normal distribution was tested with the Kolmogorov-Smirnov test. The comparison of two means was performed using a t-test for independent samples with equal or unequal variations depending on the Levene test result. Frequencies were compared with the Chi-square test. Pearson’s correlation coefficient was computed if the variables were linear and with Spearman coefficient of correlation. Multivariate linear regression was computed, enter method with the significant independent variables from the univariate analysis for each studied parameter taken as a dependent variable. Hospitalization days were analyzed as time variable with one-minus survival Cox regression in univariate and multivariate analysis, where the event was consider the recovery of the patient, opposite to transfer to intensive care unit or death. Hazard ratio (HR) and 95% confidence interval (CI) were reported. When we analyzed a qualitative variable as a dependent, we used logistic regression. All significant variables from the univariate analysis were introduced into the entered model as independent variables. We reported an odds ratio (OR) with 95% CI.

When we wanted to find the best cut-off to discriminate between cases and controls/different CT severity score values or NLR or PLR less/higher than the median, the ROC (receiver operating characteristics) curve analysis was used. The area under the ROC Curve (AUC) was reported. AUC is compared with 50% of the square area and is statistically significant if the considered parameter has the power to discriminate. The higher the rock curve is, the better the discrimination parameter we get. The cut-off was considered when the Youden index maximized, i.e., sensitivity (Se) plus Specificity (Sp). A *p*-value < .05 was taken to indicate statistical significance. The Hospital Ethics Committee approved the study.

## Results and discussion

In the present study, we analyzed 149 patients with COVID-19 and 149 age-matched healthy subjects. Baseline characteristics, clinical and blood parameters of patients with SARS-COV2 infection are shown in Table 1.

**Table 1:** Demographics and clinical characteristics of COVID-19 patients.

Parameter	COVID-19 patients (n = 149)
	Median (25th - 75th percentile)
Body mass index (BMI)	25.95 (23.02; 29.93) kg/m2
Rural	51 (34.2%)
Smoking status (pack a year index)	
1. Current smoker	28 (18.8%)
2. Former smoker	20 (13.4%)
3. Nonsmoker	101 (67.8%)
Alcohol consumption	
1. On occasions	13 (8.7%)
2. Never	87 (91.3%)
Previous antibiotic treatment	6 (4%)
Comorbidities, no. (%)	
1. Cardiovascular disease	40 (26.8%)
2. Arterial hypertension	34 (22.8%)
3. Diabetes	12 (8.1%)
4. Respiratory diseases	10 (6.7%)
5. Hypothyroidism	8 (5.3%)
Drugs	
1. Beta-blockers	21 (14.1%)
2. ACE -inhibitors	14 (9.4%)
3. Diuretics	13 (8.7%)
4. Antidepressants	13 (8.7%)
5. Sartans	5 (3.4%)
D-dimers	277 (192; 884.5) (ng/ml FEU)
Ferritin	191.4 (75.25; 509.6) (ug/L)
Lactate dehydrogenase (LDH	291 (191; 391) (U/L)
Procalcitonin	0.18 (0.13; 0.22) (ng/ml)
High resolution CT severity score*	2 (1; 2)
Hospitalization days	14 (10; 20)
No of test till RT-PCT negative results	4.5 (3; 7)

*data available for 102 patients.

The average age was 46 years old in both COVID-19 patients and control. Most of our patients were nonsmokers. Among 149 COVID-19 patients who underwent blood routine examinations on admission, most of them have abnormalities in the peripheral blood system (Table 2).

**Table 2:** Inflammatory markers in COVID 19 patients and healthy controls.

Parameter	COVID-19 patients (n = 149)	Healthy controls (n = 149)	p- value
	Median (25th - 75th percentile)	Median (25th - 75th percentile)	
**Age**	46 (34; 57.5)	46 (41; 51)	0.997
**Male, no.**	69 (79.3%)	18 (20.7%)	<0.001
**White blood cells (WBC**	5.95 X*10^3^/ul (4.89; 8)	6.42 X10^3^/ul (5.7; 7.8)	0.437
**Neutrophils**	4.01X10^3^/ul (2.94; 5.5)	3.97 X10^3^/ul (3.3; 4.86)	0.027
**Lymphocyte**	1.56 X10^3^/ul (1.2; 2.03)	2.04X10^3^/ul (1.61; 2.42)	<0.001
**Eosinophils**	1.15% (0.3; 2.1)	2.2% (1.4; 3.3)	<0.001
**Eosinophils**	0.08 X10^3^/ul (0.02; 0.15)	0.14 X10^3^/ul (0.09; 0.24)	0.001
**Platelets**	248X10^3^/ul (182.5; 297)	247.5 X10^3^/ul (210.5; 287.5)	0.931
**Neutrophils-lymphocytes-ratio (NLR)**	2.56 (1.72; 3.79)	2.11 (1.65; 2.57)	0.004
**Platelets-lymphocytes ratio (PLR)**	151.85 (112.86; 211.59)	125.84 (99.02; 155.36)	<0.001
**C-reactive protein (CRP**	3.2 (0.92; 18.6) (mg/L)	2.85 (1.5; 4.58) (mg/L)	<0.001
**Erythrocyte sedimentation rate (ESR) (/mm)**	12/mm (5.5; 30)	10/mm (6; 15)	<0.001

Patients with COVID-19 had lower white blood cells count (5.95 vs 6.42, p = 0.437), but not significantly, lower significantly statistically lymphocyte count (1.56 vs 2.04 × 109/L; p < 0.001) and lower significantly statistically eosinophil count (0.08 vs 0.14 = 4 × 109/L; p < 0.001) when compared with control group. Neutrophils (4.01 vs 3.97, p = 0.027), NLR (2.56 vs 2.11, p = 0.004), PLR (151.85 vs 125.84, p< 0.001), CRP (3.2 vs 2.85, p <0.001) and ESR (12 vs 10, p <0.001) were as expected significantly higher in the studied group when compared with healthy subjects (see Table 2). When taken inflammatory markers separately NLR has best Youden index for 2.90, sensitivity = 0.41, specificity = 0.89, AUC = 0.624, p = 0.001, PLR has best Youden index for 186, sensitivity = 0.36, specificity = 0.92, AUC = 0.648, p<0.001 while eosinophils have best Youden index for 1.05, sensitivity = 0.92, specificity = 0.49, AUC = 0.740, p< 0.001. Eo# best Youden index for 0.09, sensitivity = 0.82, specificity = 0.58, AUC = 0.719, p<0.001. The average hospital stay was 20 days, with a maximum of 35. In multivariate Cox one-minus survival analysis, longer hospitalization was associated with high weight HR = 0.982 95% CI (0.967, 0.997), p = 0.016, and a more severe disease form; low neutrophils HR = 1.026 95% CI (1.011, 1.042), p = 0.001. Out of 149, 102 had chest CT performed; the rest had only chest X-Ray. Most patients had CT lesions corresponding to a severity score of 2; four patients had normal CT, and 14 patients had a severity score of 3 with signs of distortion of the lung architecture. CT severity score was smaller among smokers 1 (1, 1) than nonsmokers 2 (2,2), p<0.001. A higher score was associated with lower lymphocytes values (r = -0.291, p = 0.009) and higher values for ESR (r = 0.321, p = 0.005), CRP (r = 0.453, p<0.001), ferritin (r = 0.491, p<0.001), and LDH (r = 0.554, p<0.001), in other words, with an important inflammatory status.

Among COVID-19 patients, 34% (46 patients) were completely asymptomatic. Asymptomatic patients were especially women (31 (67.4%) vs. 37 (42.5%), p = 0.006, with low weight (p = 0.035) and without other comorbidities, such as diabetes (p = 0.015), cardiovascular disease (p = 0.047). They have a lower CT severity score one versus 2 in symptomatic patients (p = 0.004) and lower values for ferritin, CRP, procalcitonin, and ESR Table 3).

**Table 3:** Clinical characteristics and blood tests/inflammatory markers in asymptomatic and symptomatic patients.

	Asymptomatic patients (n = 46)	Symptomatic patients (n = 87)	p- value
**Age**	47 (35; 56)	46 (33; 57.5)	0.844
**Male**	15 (32.6%)	50 (57.5%)	0.006
**Weight**	70 kg (62; 76.5)	77 kg (69; 90)	0.035
**Diabetes**	0	10 (11.5%)	P = 0.015
**Cardio-vascular disease**	7 (15.2%)	37 (31%)	P = 0.047
**White blood cells (WBC)**	6.25X10^3^/ul (5.23; 8.65)	5.77 X10^3^/ul (4.64; 6.79)	0.017
Neutrophils (*)	4.1 X10^3^/ul (3; 6.31)	3.77X10^3^/ul (2.95; 4.77)	0.691
Lymphocytes(*10^**3**^**/ul)**	1.76 X10^3^/ul (1.3; 2.19)	1.49X10^3^/ul (1.17;1.96)	0.176
**Eosinophils (%)**	1.3% (0.85; 2)	1 (0.3; 2.1)%	0.650
Eosinophils (*103/ul)	0.08 X10^3^/ul (0.06; 0.19)	0.08 X10^3^/ul (0.06;0.19)	0.051
**Platelets**	257.5X10^3^/ul (183; 299)	240 X10^3^/ul (181.5; 292.5)	0.857
**NLR**	2.63 (1.83; 3.79)	2.53 (1.68; 3.87)	0.415
**PLR**	133.68 (103.74; 201.12)	165.52 (112.86; 232.1)	0.244
**C-reactive protein**	1.75 mg/dl (0.24; 11.75)	5.35 mg/dl (1.5; 43.25)	0.003
**ESR**	10mm/h (4.5; 22.5)	15.5mm/h (6; 32)	0.536
**Procalcitonin**	0.14 ng/ml (0.05; 0.18)	0.19 ng/ml (0.15; 0.22)	0.010
**Ferritin**	127.7 ng/ml (47.85; 267.05)	193.9 ng/ml (93.25; 526)	0.023
**LDH**	232 U/L (170; 295.5)	321.5 U/L (235.5; 467.5)	0.001
**D-dimers**	235mcg/ml (194; 343)	265 mcg/ml (170; 511)	0.786
**CT severity score**	1(1;2)	2 (1;2)	0.004

In the multivariate analysis (logistic regression) with the significant variables from the univariate analysis as independent variables and with the symptomatic variable as the dependent variable, the gender OR = 3.63, 95% CI (1.38; 9.50), p = 0.009 and low level of leukocytes OR = 0.794, 95% CI (0.64; 0.99), p = 0.037 remained significant. Both NLR and PLR correlated positive chest CT scan severity (Table 4). When NLR value is below 5.04, CT score is lower than 3 with a probability of 94%, while when NLR is higher than 5.04, the probability of CT changes is only 50%. For eosinophils, a value of 0.35% corresponds to chest CT severity of 2 (Se = 0.88, Sp = 0.43, AUC = 0.661, 95% CI (0.544; 0.779), p = 0.021. A negative correlation of NLR was observed with eosinophils (Table 4) and with BMI.

**Table 4:** NLR and PLR correlation.

	Parameter	Coefficient of correlation Spearman	p	B (95% CI for B) in multivariate analysis	p
**Neutrophils-to-lymphocytes ratio (NLR)**	Age	0.33	0.001		
	BMI	0.28	<0.001		
	EO%	-0.28	0.001		
	EO absolute value	-0.25	**0.004**		
	ESR	0.38	<0.001		
	CRP	0.51	<0.001	0.034 (0.022;0.046)	<0.001
	Ferritin	0.44	<0.001		
	LDH	0.39	<0.001		
	Procalcitonin	0.46	<0.001 (n = 54)	1.873 (0.244; 3.502)*	0.026
	D-Dimers	0.49	<0.001 (n = 49)		
**Platelets-to-lymphocytes ratio (PLR)**	Age	0.40	<0.001		
	ESR	0.52	<0.001	0.924 (0.074; 1.774)	0.033
	CRP	052	<0.001		
	Ferritin	0.31	0.001		
	LDH	0.36	<0.001		
	Procalcitonin	0.47	<0.001 (n = 54)		
	D-Dimers	0.36	0.011 (n = 49)		

Analyzing patients’ smoking status, there was a statistically significant difference in NLR and PLR. NLR had a value 2.15 (1.61; 3.53) among nonsmokers, 3.14 (2.58; 4.16) among ex-smokers and 2.41 (1.82; 3.17) in smokers (p = 0.046). PLR was 151.85 (115.29; 204.9) in COVID-19 that never smoked, 170.82 (131.21; 236.4) in former smokers and 128.33 (95.58; 181.92) in active smokers. PLR was also statistically significantly different (p = 0.003) between patients with previous antibiotic treatment 229.68 (175.28; 292.73) when compared with those without treatment 146.31 (107.26; 192.24).

The optimal cut-off values calculated by the ROC analysis and areas under the curve (AUC) of age, NLR, and PLR were also evaluated. The age from which NLR is above median values of 2.56 is 53 years old (AUC = 0.690, 95%CI (0.603–0.777), p<0.001, Se = 51%, Sp = 86%), while for PLR is 151.85 at 46.5 years (AUC = 0.687, 95%CI (0.600–0.773), p<0.001, Se = 63%, Sp = 68).

As expected, in our study, inflammation was important in COVID-19 patients, and alongside other inflammatory markers, NLR and PLR had significantly higher values in these patients. In our study a NLR = 2.90 and PLR = 186 have a good specificity (0.89, p = 0.001, respectively 0.92, p<0.001) in patients with SARS-COV2 infection. If in other inflammatory diseases (COPD, sleep apnea, myocardial infarction), PLR does not have a strong correlation with other inflammatory markers, in COVID-19 patients, the correlation is even stronger than in NLR, suggesting the importance of coagulation disturbances.

As pneumonia in COVID-19 is secondary to inflammatory processes, we sought to look for a correlation between inflammatory markers and CT changes’ severity. In our study, more significant severity CT changes correlate with NLR, PLR, and eosinophils. Several studies have demonstrated the presence of CT changes in asymptomatic patients [19]. The aim of this study to evaluate the correlation between inflammatory status markers (neutrophil-to-lymphocyte ratio (NLR), platelets-to-lymphocytes ratio (PLR), eosinophils (EO), ferritin, D-dimers, procalcitonin) with the severity of CT lesions in COVID-19 patients was reached. 27% (41) of our patients had cardiovascular disease (CVD), especially hypertension. CVD is a well-known risk factor, and very few of them have previously been treated with ACR inhibitors.

In the beginning, when the patients generally experience non-specific symptoms, peripheral blood tests (leukocyte, lymphocytes, platelets) might be in the normal range or slightly reduced. Lymphocytopenia seems to be the most frequent manifestation as it appears in up to 83.2% of patients, whereas only 33.7% had leukopenia [20]. Eosinophils tend to have smaller values from the beginning. Once the systemic inflammatory markers reach critical values ("cytokine storm"), significant lymphopenia becomes evident, and eosinopenia aggravates [21, 22].

In the present study, patients with COVID-19 had clinically significant lower values for leukocytes, lymphocytes, eosinophils, CRP, and ESR compared with the control group. An eosinophils value of 0.09 *103ug/ml seems to be suggestive for SARS-COV2 infection (Se = 82%, Sp = 58%, AUC = 0.719, p<0.001).

Xie and colleagues reported an eosinophils cut-off of 0.15 with an AUC of 0.74. The difference might be due to patient selection as their studies included more severe patients (in Romania was compulsory the hospitalization of asymptomatic patients). NLR and PLR had higher values in COVID-19 group when compared with control (2.56 vs 2.11, p = 0.004respectively 151.85 vs 125.84, p<0.001). For NLR the cut-off value found was 2.90 with a Se = 41%, Sp = 89%, AUC = 0.624, p = 0.001. PLR was 186 with a Se = 36%, Sp = 92%, AUC = 0.648, p< 0.001. As expected, NLR and PLR were higher among former and current smokers than among never smokers. Most of our patients were never smokers 101(57.8%). NLR and PLR also correlated with age and already established inflammatory markers such as ESR, CRP, ferritin, procalcitonin, and d-dimers.

Reported cut-off values were 3.3 and 180 for NLR, and PLR, respectively. The highest Sp and Se were 63.6% and 88%, 44% and %77 for NLR, and PLR, respectively, similar to ours.

CRP and ESR were elevated and correlated positively with NLR and PLR. D-dimers had a value = 277mcg/ml, while ferritin was 191.4 ng/ml. They both correlated positively with PLR and NLR. Data found are similar to those existing in the literature [23].

Guan and colleagues reported that non-survivors, as compared with survivors, presented more often with high LDH value, increased procalcitonin (OR = 4.76; 95% CI: 2.74–8.29, I2 = 34%), and high ferritin level and D-dimers [24, 25]. Neutrophilia and eosinophilia are almost always present and tend to be associated with worse prognosis. This might suggest according to Sun and colleagues [26], that neutrophils and eosinophils contribute to cytokine release. More than the exact value itself, it is more critical the variation of this parameter.

Interestingly, PLR has in COVID -19 patients a better correlation with classical inflammation makes than in other diseases. This could be the consequence of coagulation changes that appear in COVID-19 [22, 26, 27]. Peripheral bloodcells, eosinophils, in particular, seem to help predict severe disease as they tend to accumulate in infected tissues "to fight" the virus. This leads to decrease values in peripheral blood. All COVID-19 patients, regardless of their severity, had low eosinophile values in the peripheral blood. The timeframe for eosinophils to recover was larger for patients admitted in the ICU departments. In these patients, eosinophil count gradually increased after the seventh day of admission. It is also worth mentioning that pthat the delay in eosinophil recovery was associated with increased risk of the severe outcome of COVID-19 after age adjustment (odds ratio, 2.291). Therefore we have to consider the fact that persistent low eosinophil counts might be an ominous sign of severe disease. More than the punctual values themselves, the dynamic observation of blood routine parameters seems to have essential value for predicting disease progression and early warning of clinical type changes [28].

Compared to other differential blood parameters, in COVID-19, patients’ eosinophils and basophils have extremely low values. They have even lower values than in general infections. When focusing on eosinopenia, Du and colleagues found it in almost every patient who died from COVID-19 [29, 30]. Eosinopenia is also more frequent in positive COVID-19 patients, compared with negative COVID-19 patients (78.8% versus 35.8%) [24, 30, 31]. Zhaoand colleagues suggested that eosinopenia could be a reliable factor for diagnosis when combined with lymphopenia [2, 31]. In the present study, absolute counts were used, and positive COVID-19 patients had an average eosinophil count of 0.02 × 109/L, while in the negative COVID-19 group, eosinophils were 0.05 × 109/L. Although eosinopenia and basophilia have been reported, a definitive conclusion is difficult to draw with current equipment due to a lack of sensitivity for this cell’s small concentrations. Why these parameters tend lower than the normal values need further investigation [30–32]. Among the study’s limitations, we must underline the lack of disease stratification (there has not been any clinical assessment of the severity), study design, and size sample. Nevertheless, as this topic’s information is scarce, we thought this to be a good starting point for future clinical trials.

## Conclusions

This cross-sectional study focuses on hematological parameters in patients with COVID-19 disease when compared with healthy subjects. It evaluates several accessible and widely uses inflammatory markers (neutrophils, lymphocytes, eosinophils, NLR, PLR, D-dimers, and ferritin) in COVID-19 patients. Some markers: NLR, PLR, and eosinophils, are determined in both COVID-19 patients and age-matched healthy subjects, offering some cut-off values that could guide us in when to suspect COVID-19. NLR and PLR have proven to be reliable markers in several diseases that go with systemic inflammation. As shown, they have a higher value in COVID 19 patients, and even more, they correlated with classical inflammatory markers such as CRP, ESR, and with those specific to SARS-cov2 infection. A value of 2.8 for NLR and 180 for PLR seem to be suggestive for COVID-19 and eosinophils = 0.15. As COVID-19 pneumonia is secondary to inflammation, we showed that a more severe inflammation, as evaluated through the markers mentioned earlier, correlates with more severe lesions on thorax CT. There are not many data concerning this subject. We thought this to be an important one as there is not always a correlation between clinical symptoms and CT changes, as it has been shown that up to 50% of COVID -19 asymptomatic patients could have CT changes. Higher levels in NLR, PLR should prompt the clinician to prescribe a thorax CT as it could reveal essential lesions that could influence the patient’s future management.

## Supporting information

S1 Data (XLSX)

## Abbreviations

WBC: White blood count cell
NLR: neutrophil-to-lymphocyte ratio
PLR: platelet-to-lymphocyte ratio
CRP: C-reactive protein
LYM: lymphocyte
NEU: Neutrophils
EO: Eosinophils
PCT: procalcitonin
